# New Sight of Intelligent Algorithm Models and Medical Devices in Bioengineering: Updates and Directions

**DOI:** 10.3390/bioengineering12050455

**Published:** 2025-04-25

**Authors:** Luca Mesin

**Affiliations:** Mathematical Biology and Physiology, Department of Electronics and Telecommunications, Politecnico di Torino, 10129 Turin, Italy; luca.mesin@polito.it; Tel.: +39-011-0904085

The integration of artificial intelligence (AI) models and advanced medical devices has led to significant advancements in healthcare, rehabilitation, and biomedical research. This Special Issue aims to highlight recent research and technological developments in this interdisciplinary field. The selected contributions explore various approaches, including intelligent algorithms for medical diagnosis, rehabilitation robotics, and optimization techniques for biomedical applications.

This Special Issue comprises five articles that investigate innovative methods in bioengineering. Below is a summary of each contribution.

**Human-Aware Control for Physically Interacting Robots**

This paper introduces a predictive model for human movement, incorporating different levels of the human sensorimotor system [1]. A nonlinear model of predictive control is used to enhance human–robot interaction. The developed prediction of human movement is performed by a holistic model, based on neuroscientific and biomechanical theories of human motor control. The following levels of the human sensorimotor system hierarchy are included: high-level decision-making by internal models, muscle synergies, and physiological muscle mechanics. Arm kinematics and neuromuscular activities are estimated efficiently, making the model suitable for repetitive simulations within an algorithm for robot control, to predict the user’s behavior in human-robot interactions. Specifically, based on the proposed nonlinear predictive framework, a human-aware control algorithm is implemented, which internally runs simulations to predict the user’s interactive future patterns of movement. Consequently, it can optimize the robot’s motor torques to minimize the user’s neuromuscular effort. Simulation results of a two-link robot arm are presented. The model replicates salient features of human movements, and the human-aware controller demonstrates the ability to predict and minimize the user’s neuromuscular effort in a collaborative task.

As robots become more integrated into human environments, ensuring safe and intuitive interaction with people is increasingly important. Additional recent methods improving human–machine interaction include myoelectric control [2] and the brain–computer interface [3].

**Intelligent Evaluation Method for Scoliosis Using Mobile-Based Back Photos**

The paper proposes a non-invasive method for scoliosis evaluation using back images taken by a mobile phone [4]. Scoliosis is a common condition in adolescents [5], and early detection and intervention are crucial [6]. The traditional scoliosis examination based on X-ray film is not suitable for large-scale screening and dynamic evaluation during rehabilitation. This study proposes an evaluation method for scoliosis using back photos taken by mobile phones, involving three main aspects: (1) an algorithm for judging the type of spinal coronal curvature based on the key point detection model of YOLOv8, (2) a method for evaluating the coronal plane of the spine based on the key points of the human back, and (3) a measurement algorithm of trunk rotation based on multi-scale automatic peak detection. A public dataset and clinical paired data (mobile phone photo and X-ray) are used for testing. The results show high accuracy and effectiveness in distinguishing the type of spinal curvature and evaluating the degree of deviation, surpassing other deep learning algorithms. The tool is promising for the early detection of spinal asymmetry, suggesting the potential for at-home scoliosis monitoring.

**Deep Learning-Based Forensic Age Estimation**

This paper presents a deep learning model for forensic age estimation [7]. This research contributes to the growing application of AI in forensic science. Dental age estimation is extensively employed in forensic medicine practice [8]. However, the accuracy of conventional methods fails to satisfy the need for precision, particularly when applied to adults. This study proposes an approach for age estimation utilizing orthopantomograms (OPGs). A new dental dataset comprising OPGs of 27,957 individuals, covering an age range from newborn to 93 years, was used. The age annotations were verified using ID card details. Many neural network details were analyzed to accurately estimate age, such as optimal network depth, convolution kernel size, multi-branch architecture, and early layer feature reuse. Two sets of models are proposed: one exhibits superior performance (AGENet) and the other is lightweight (AGE-SPOS). They demonstrate remarkable results, with AGENet and AGE-SPOS achieving a mean absolute error of 1.70 and 1.80 years, respectively, outperforming traditional age estimation techniques.

**Clustering Fibromyalgia Patients Using Syntactic and Semantic Distance Measures**

Fibromyalgia is a common chronic pain disorder that presents diagnostic challenges for clinicians [9]. This paper proposes a novel approach to clustering fibromyalgia patients based on syntactic and semantic distance measures [10]. Fibromyalgia is a prevalent condition characterized by chronic widespread musculoskeletal pain, fatigue, and sleep disturbances. Identifying patient subgroups can assist in understanding the modifiable risk factors associated with each cluster and optimize personalized therapeutic strategies. The study recruited 1370 fibromyalgia patients. The methodology, referred to as CDI-SSV (Clustering, Distance measures, and Iterative Statistical and Semantic Validation), integrates various clustering algorithms (including K-means, Gaussian mixture, and agglomerative clustering) and validation techniques to identify the optimal number of clusters. The results suggest three distinct severity levels of fibromyalgia, validated through machine learning models. Using multi-dimensional validation, the proposed method improves patient stratification, aiding personalized treatment strategies.

The CDI-SSV methodology offers significant potential for improving the classification of complex patients, providing clinical markers for the personalized diagnosis, management, and prognosis of fibromyalgia patients.

**Auto-Tuning Algorithm for Mass Spectrometry using Particle Swarm Optimization**

Quadrupole mass spectrometers (QMSs) are widely used for clinical diagnosis, chemical analysis, and environmental monitoring due to their high sensitivity and selectivity [11]. Traditional tuning techniques are manual and time-consuming and rely on skilled engineers. The objective of this study is to develop an auto-tuning algorithm to improve the automation and efficiency of QMSs. The paper proposes an improved particle swarm optimization (PSO) algorithm for tuning the parameters of the QMS [12]. The PSO algorithm uses particles to find optimal solutions by adjusting their velocity and position. Enhancements include simulated annealing, dynamic boundaries, and multi-inertia weights to prevent premature convergence and local optimal solutions. The improved PSO algorithm is combined with QMS-specific tuning requirements, focusing on mass axis calibration, resolution adjustment, and the optimization of lens and ion source parameters. The technique enhances detection sensitivity and resolution, demonstrating significant improvements in analytical instrumentation for bioengineering applications and reducing the tuning difficulty.

In conclusion, this Special Issue shows significant advances in AI-based medical applications and device optimization. The papers presented highlight how intelligent algorithms are revolutionizing healthcare research and applications, opening up new perspectives for future innovations.
